# Heterostructured Bi_2_O_3_@rGO Anode for Electrochemical Sodium Storage

**DOI:** 10.3390/ma15082787

**Published:** 2022-04-11

**Authors:** Benrong Hai, Changsheng Liu

**Affiliations:** School of Materials Science and Engineering, Northeastern University, Shenyang 110819, China; hbr1019@163.com

**Keywords:** sodium-ion battery, bismuth oxide, alloy, reduced graphene oxide, coulombic efficiency

## Abstract

Bismuth oxide (Bi_2_O_3_) is an auspicious anode material for sodium-ion batteries owing to its high theoretical capacity and abundant Bi resources. However, the poor electronic conductivity and huge volume expansion of Bi_2_O_3_ during cycling lead to the low coulombic efficiency and unstable cycling stability. Aiming to suppress these issues, we use highly conductive reduced graphene oxide (rGO) as a continuous skeleton to fabricate a Bi_2_O_3_@rGO heterostructure. It exhibits high reversibility and stability for electrochemical sodium storage by delivering a reversible capacity of 161 mAh g^−1^ after 100 cycles at 50 mA g^−1^, which completely outperforms Bi_2_O_3_ (43 mAh g^−1^). In addition, the coulombic efficiency of the heterostructure stabilizes at >90% upon only 3 cycles. The results can be attributed to the dual function of rGO in supporting Bi_2_O_3_ nanoparticles and providing conductive pathways to fasten electron transport.

## 1. Introduction

Energy storage technologies are of high importance to meet currently flourishing energy demand and environmental pollution consequences depletion of fossil fuels. Lithium-ion batteries (LIBs), owing to high-energy densities and excellent long-term cycle life, have been main energy storge medium in the past decade [1]. LIBs show great performance and dominate the market of portable electronics, but the limited resources and uneven global distributions of lithium have raised serious concern on the sustainability of the LIB technology [2]. Thus, it is highly urgent to research new energy storage systems based on more abundant elements. Sodium-ion batteries (SIBs) have focused global attention since Na is widely distributed and SIBs exhibit competitive performance to LIBs. In particular, SIBs are cost-competitive to meet future large-scale energy storage requirements [3]. However, in comparison with lithium ion, sodium ion has a larger ionic radius (1.02 Å), it’s more arduous challenges to develop appropriate materials for reversible and fast sodium ion insertion/extraction. And for the anode materials, huge volume expansion and sluggish kinetics during cycling process may result in fast capacity attenuation and low coulombic efficiency [4].

In recent decades, a large amount of effort has been made to develop brillant anode materials. Carbonaceous materials [5,6], metal sulfides [7,8], metal oxides [9,10] and metal phosphides [11,12] have been extensively researched as anodes for SIBs. Among them, alloy-based metal oxides are attractive as anode materials for SIBs, due to low redox potential, high volumetric energy density and theoretical specific capacity [13]. Bi_2_O_3_ has been reported as an auspicious anodes owing to its high gravimetric specific capacity, abundant resources and environmental sustainability [14]. Deng et al. synthesized bismuth oxide/reduced graphene oxide nanocomposites as an electrode for LIBs, which deliver a capacity of 347.3 mAh g^−1^ with 79% capacity retention (after 100 cycles at 600 mA g^−1^) [15]. Kim et al. prepared Bi_2_O_3_/carbon composites as an anode for SIBs by simple ball-milling method. The Bi_2_O_3_/carbon electrode exhibited a high capacity of 440 mAh g^−1^ after 20 cycles at 714.3 mA g^−1^ [16]. However, metal oxides still suffer from inherent problems of unsatisfactory electronic conductivity, sluggish Na-ion transfer rate and severe volume expansion during cycling processes [17]. To address these issues, many optimized architectures have been constructed by researchers, such as hybridizing with carbonaceous matrix [18,19], compositing with different materials [20] and designing appreciated structures [21,22]. Among these, the heterostructure constructed by active material and carbonaceous substrate is one of the most promising structures for SIBs [23]. RGO stands out among various carbonaceous matrices because of its fast electron mobility, ideal large specific surface area and high charge carrier mobility. It was reported that alloy-based metal oxide@rGO composite electrodes have significantly improved the electrochemical performance of SIBs [24]. For example, 3D SnO_2_@rGO composites with a large amount of internal void space are prepared to resolve the huge volume deformation problem of SnO_2_-based materials as anodes for SIBs [25]; the Fe_2_O_3_/holey rGO anode for SIBs exhibits excellent cyclability and rate capability owing to the introduction of wrinkled rGO, since wrinkles of graphene layers act as a template for anchoring Fe_2_O_3_ nanoparticles and effectively relax the strain induced by the volume deformation [26]. 

**Figure 1:**
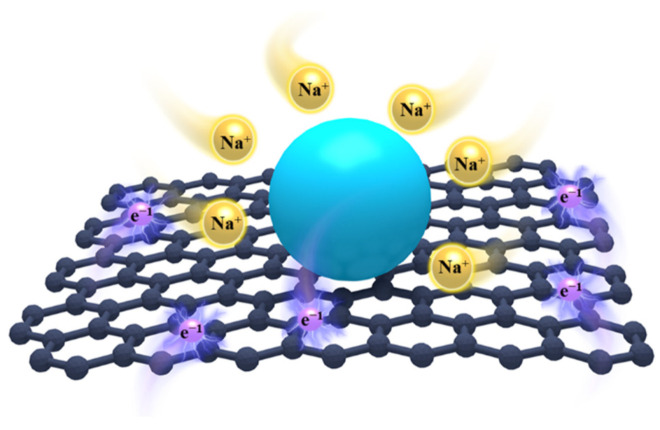
Schematic of Na-ion and electron transfer in Bi_2_O_3_@rGO.

In this work, we report that the Bi_2_O_3_@rGO heterostructure exhibits drastically enhanced sodium storge in comparison with bare Bi_2_O_3_ particles. As shown in Figure 1, Bi_2_O_3_ nanoparticles are anchored on the rGO layer with a large surface area. This enables the shortening of the Na-ion diffusion length and increases the contact with electrolyte. Meanwhile, rGO layers with high electrical conductivity can improve electron diffusion and serve as a skeleton to prevent the shedding or agglomeration of Bi_2_O_3_ nanoparticles during cycling. As a result, the Bi_2_O_3_@rGO heterostructure electrode exhibits an excellent coulombic efficiency (EC) reaching > 90% after 3 cycles and a good cycling ability of 160.9 mAh g^−1^ after 100 cycles at 50 mA g^−1^. 

## 2. Materials and Methods

### 2.1. Synthesis of Bi_2_O_3_@rGO

Modified Hummers method was adopted to prepare graphene oxides (GO) [27,28]. The Bi@rGO precursor was synthesized by a typical hydrothermal method. GO (50 mg) was ultrasonically dispersed in N,N-dimethylformamide (40 mL), followed by adding Bi(NO_3_)_3_·5H_2_O (100 mg) and polyvinylpyrrolidone (300 mg) into the dispersion, then stirred until completely dissolved. The turbid liquid was moved to a Teflon-lined stainless-steel autoclave and heated (180 °C, 8 h). After cooling down, the obtained precipitate was washed and dried. The Bi_2_O_3_@rGO heterostructure was obtained by a further annealing treatment of the resulting precursor at 300 °C for 4 h (5 °C min^−1^) in ambient air. Bi_2_O_3_ particles were prepared under the same conditions without GO.

### 2.2. Structural Refinement

X-ray diffraction (XRD) was performed on a Bruker-axs Discover D8 (Rigaku, Japan) with Cu Kα (1.54056 Å). Renishaw Raman Spectrometer (HORIBA, Kyoto, Japan) employing visible excitation at 532 nm was used to record the Raman spectrum. X-ray photoelectron spectroscopy (XPS) was measured with a Thermo Fisher Nexsa X-ray photoelectron spectrometer (Thermo Fisher, Waltham, MA, USA), which is equipped with a monochromatic Al Ka X-ray (1486.6 eV). Scanning electron microscopy (SEM) images were obtained by utilizing a Hitachi S4800 (Hitachi, Japan). Transmission electron microscopy (TEM) was achieved on a JEOL 2100F (JEOL, Kyoto, Japan) transmission electron microscope. 

### 2.3. Electrochemical Investigation

Electrodes were prepared by dispersing Bi_2_O_3_@rGO (80 wt%), Super-P (10 wt%) and polyvinylidene fluoride dissolved in N-methylpyrrolidinone (10 wt%). The obtained slurry was coated on a copper foil, pressed and vacuum-dried at 110 °C, and the mass loading was around 1 mg cm^−2^. Electrochemical tests were performed using CR2032 coins, which were assembled in a nitrogen-filled glovebox (oxygen and moisture concentrations < 0.1 ppm). A glass microfiber filter (pore size ~1 μm) was employed to separate the sodium metal disk counter electrode from the electrode. A tatal of 1 M sodium perchlorate in ethylene carbonate/propylene carbonate (1:1) was prepared as electrolyte. Electrochemical impedance spectroscopy (EIS) and cyclic voltammetry (CV) were measured on a Bio-Logic VSP electrochemical workstation, and the CVs were between 0.01–2.5 V (vs. Na^+^/Na). Galvanostatic charge-discharge was measured on a Land battery testing system (CT2001A, Land, China) in a potential range of 0.01–2.5 V (vs. Na^+^/Na). The EIS was carried out with an amplitude of 5 mV at open-circuit voltage and the frequency range was 10 mHz–100 kHz.

## 3. Results

### 3.1. Morphology, Structure and Composition Analysis

**Figure 2:**
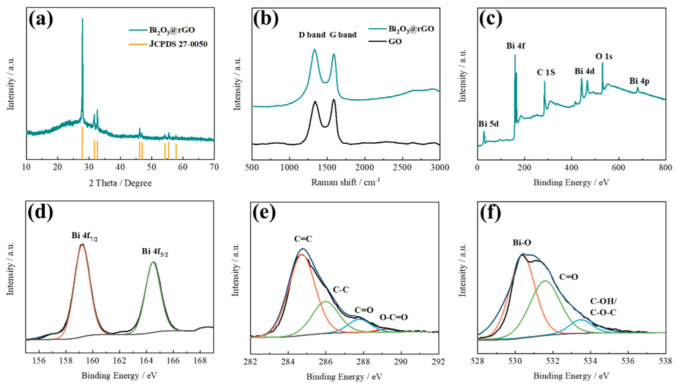
(**a**) XRD pattern of Bi_2_O_3_@rGO. (**b**) Raman spectra of Bi_2_O_3_@rGO and GO. (**c**) XPS survey of Bi_2_O_3_@rGO. High resolution XPS spectra for (**d**) Bi, (**e**) C and (**f**) O elements of Bi_2_O_3_@rGO.

**Figure A1:**
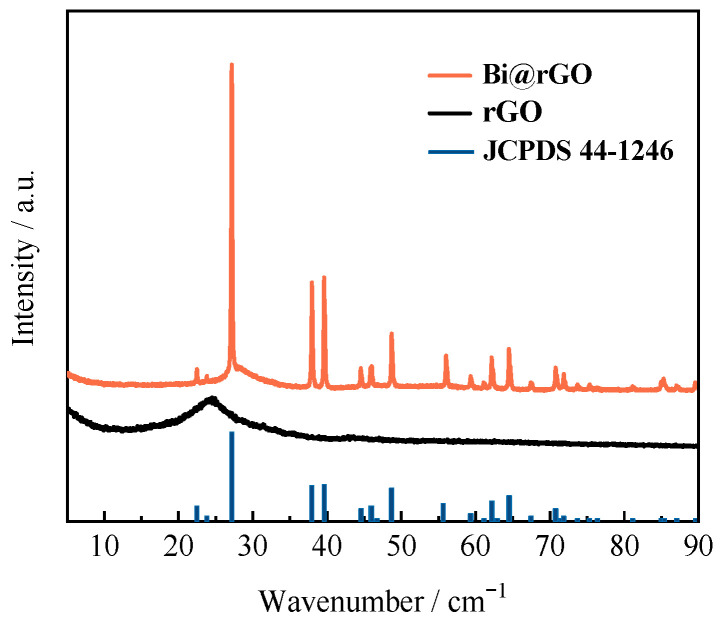
XRD patterns of Bi@rGO, rGO and JCPDS 44-1246 (rhombohedral Bi).

**Figure A2:**
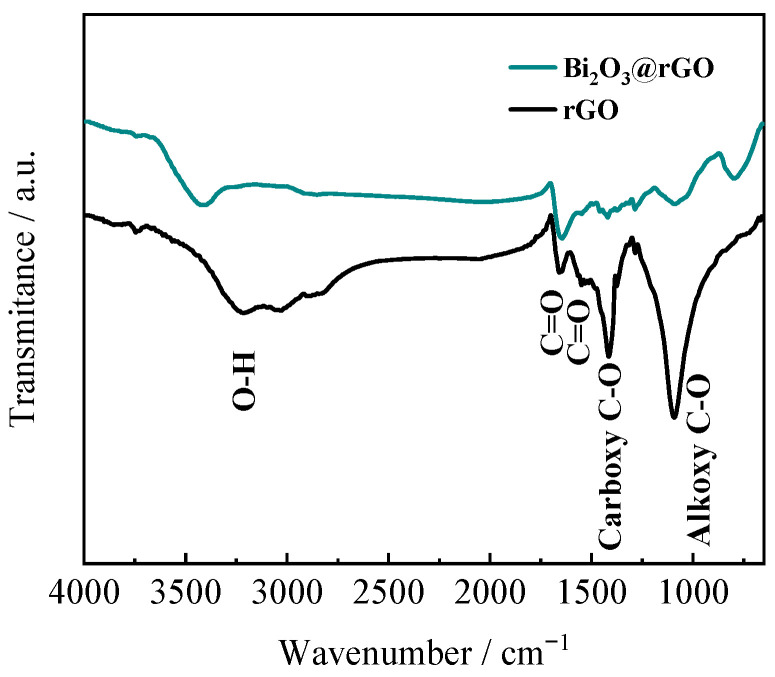
FTIR spectra of Bi_2_O_3_@rGO and GO.

Crystalline structure and phase purity of Bi_2_O_3_@rGO heterostructure are characterized by XRD. The XRD pattern of Bi_2_O_3_@rGO (Figure 2a) matches well with the peaks of tetragonal δ-Bi_2_O_3_ (JCPDS 27-0050) at 2θ = 27.9° (201), 31.8° (002), 32.7° (220), 46.2° (222), 46.9° (400), 54.3° (203), 55.5° (421), and 57.8° (402). It has been reported that δ-Bi_2_O_3_ exhibits the fastest oxygen ion conduction in the six polymorphs of Bi_2_O_3_ [29]. No peaks of Bi (Figure A1) are observed, suggesting high purity of the material, and that Bi has been completely converted to Bi_2_O_3_ after annealing treatment [30,31]. Figure 2b displays the Raman spectra of Bi_2_O_3_@rGO and GO. The D band (1350 cm^−1^) and G band (1590 cm^−1^) confirm the presence of rGO in the composite. The surface chemistry and interaction between Bi_2_O_3_ and the rGO layers are further investigated by XPS measurements. The existence of Bi, C and O elements are indicated in Figure 2c. The high-resolution XPS spectrum (Figure 2d) for Bi 4f exhibits two peaks located at 158.9 and 164.2 eV, attributed to the Bi 4f_7/2_ and Bi 4f_5/2_, respectively. In Figure 2e, the C 1s spectrum of Bi@rGO shows four peaks of sp^2^ C (284.8 eV), C-C (285.9 eV), C=O (287.8 eV) and O-C=O (289.3 eV) [14]. The C 1s spectrum is consistent with the FTIR result in Figure A2. The peaks for O1s could be fitted into three sub peaks at about 530.4, 531.8 and 533.6 eV (Figure 2f), which correspond to the Bi-O band, carbonyl and hydroxyl/epoxy, respectively [18].

**Figure 3:**
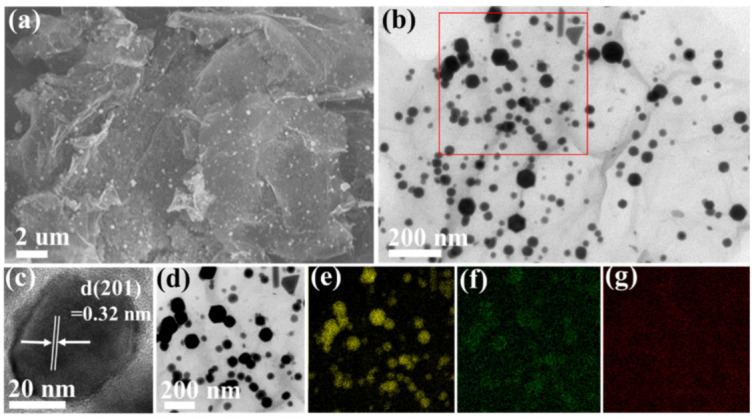
(**a**) SEM image of Bi_2_O_3_@rGO. (**b**) TEM and (**c**) high-resolution TEM images of Bi_2_O_3_@rGO. (**d**) The selected region in (**b**) and EDS mapping of (**e**) Bi, (**f**) O, (**g**) C.

**Figure A3:**
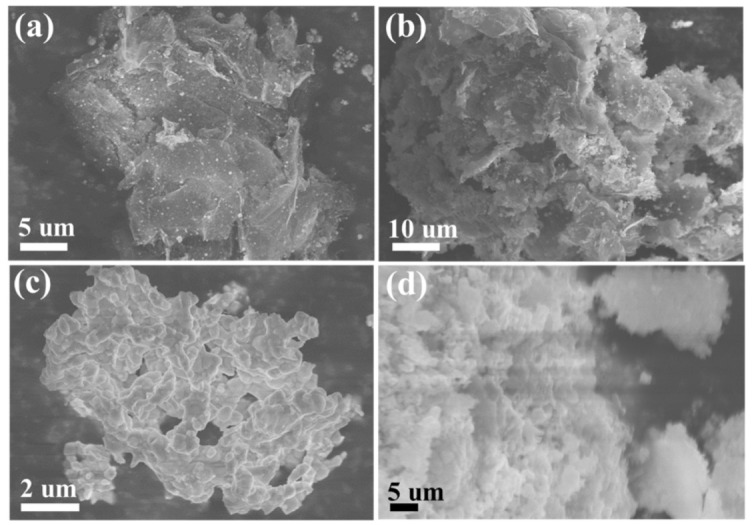
SEM of (**a**) Bi_2_O_3_@rGO, (**b**) Bi@rGO, (**c**) Bi_2_O_3_ and (**d**) Bi.

Figure 3a and Figure A3a represent the SEM images of Bi_2_O_3_@rGO where Bi_2_O_3_ nanoparticles are well anchored on rGO layers without agglomeration, presumably due to electrostatic repulsion that keeps sufficient space between Bi_2_O_3_ nanoparticles. The SEM images of Bi_2_O_3_@rGO show similar morphology features to that of Bi@rGO (Figure A3b), indicating that the anneal process at 300 °C in ambient air does not damage the morphologies of eitherBi_2_O_3_ nanoparticles orrGO layers. In contrast, bare Bi_2_O_3_ particles (Figure A3c) agglomerate without rGO, forming clusters with a size of multiple micrometers after annealing from Bi particles (Figure A3d). The structure of Bi_2_O_3_@rGO is further manifested by TEM image (Figure 3b). Bi_2_O_3_ nanoparticles are uniformly distributed on rGO, and the wrinkles of rGO can be clearly seen, indicating that the layers are thin. Once again, the agglomeration of Bi_2_O_3_ nanoparticles is effectively prevented with the presence of rGO and the size of Bi_2_O_3_ nanoparticles in the composite is less than 100 nm. The high-resolution TEM image in Figure 3c exhibits that the lattice spacing is 0.32 nm, corresponding to the (201) plane of δ-Bi_2_O_3_. The EDS mapping of the selected region (indicated by the red frame in Figure 3b) is shown in Figure 3e–g, which further demonstrates the successful fabrication of the Bi_2_O_3_@rGO composite.

### 3.2. Sodium Storage Behavior

**Figure 4:**
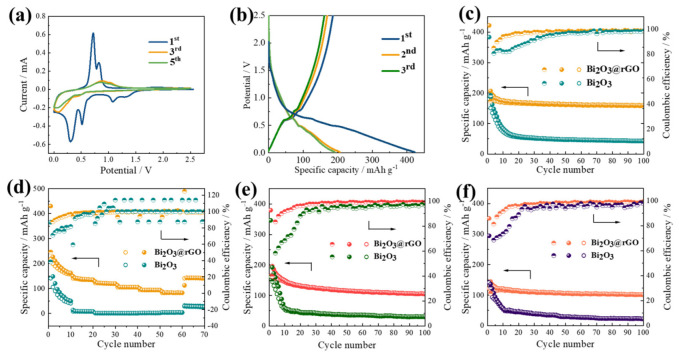
(**a**) CV curves and (**b**) discharge-charge profiles of Bi_2_O_3_@rGO. (**c**) Cycle performance of Bi_2_O_3_@rGO and Bi_2_O_3_ at 50 mA g^−1^. (**d**) Rate performance of Bi_2_O_3_@rGO and Bi_2_O_3_ at 50 mA g^−1^. Cycle performance at (**e**) 200 mA g^−1^ and (**f**) 1A g^−1^.

**Figure A4:**
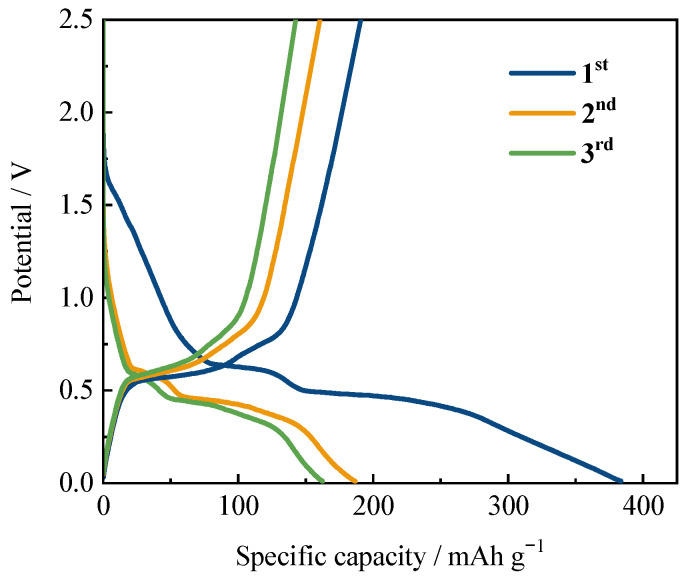
Charge-discharge profiles of Bi_2_O_3_.

The CV curves of the composite are shown in Figure 4a. The peak observed at 1.06 V in the first cathodic scan corresponds to the reduction of Bi_2_O_3_ to Bi and the formation of SEI film. The peak at 0.52 V is attributed to the alloy reaction process from Bi to NaBi and the peak located at 0.30 V corresponds to NaBi to produce Na_3_Bi [32]. The peaks at 0.72 V and 0.82 V are caused by the desodiation of Na_3_Bi → NaBi → Bi [33]. The overlapping curves of the third and fifth cycles reveal the good reversibility of the composite. Figure 4b exhibits the initial three discharge-charge profiles of Bi_2_O_3_@rGO at 50 mA g^−1^ and the initial discharge capacity is 421.8 mAh g^−1^. Two discharge plateaus (0.52 and 0.30 V) and two charge plateaus (0.72 and 0.82 V) are in good agreement with the CV curves of Bi_2_O_3_@rGO. The initial three charge-discharge profiles of Bi_2_O_3_ at 50 mA g^−1^ (Figure A4) show an initial discharge capacity of 383.6 mAh g^−1^. As seen, the discharge capacities at the second and third cycles are 186.8 and 162.5 mAh g^−1^, respectively. This suggests that the Bi_2_O_3_ electrode without rGO has a mass of irreversible capacity in the first two discharge–charge cycles. In comparison with Bi_2_O_3_, the Bi_2_O_3_@rGO electrode shows better capacity retention in the early cycles. The fact that the introduction of rGO can effectively mitigate the rapid capacity decay is also reported by other studies [32,34].

**Figure A5:**
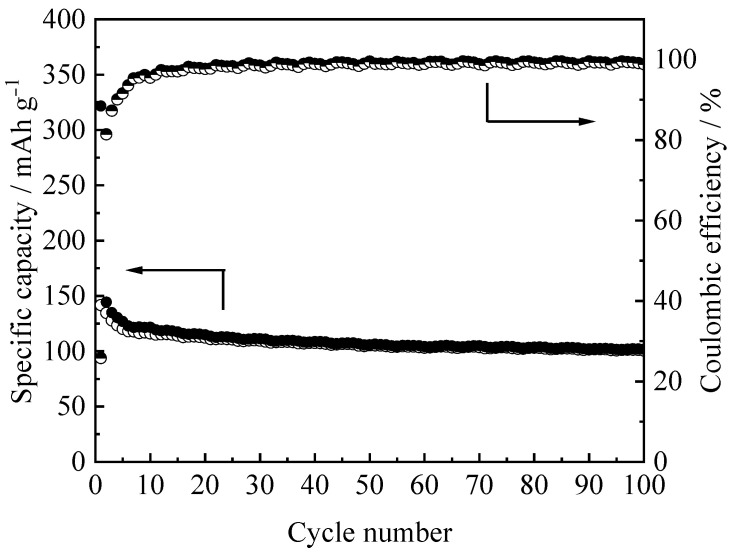
Cycle performance of rGO at 50 mA g^−1^.

**Figure A6:**
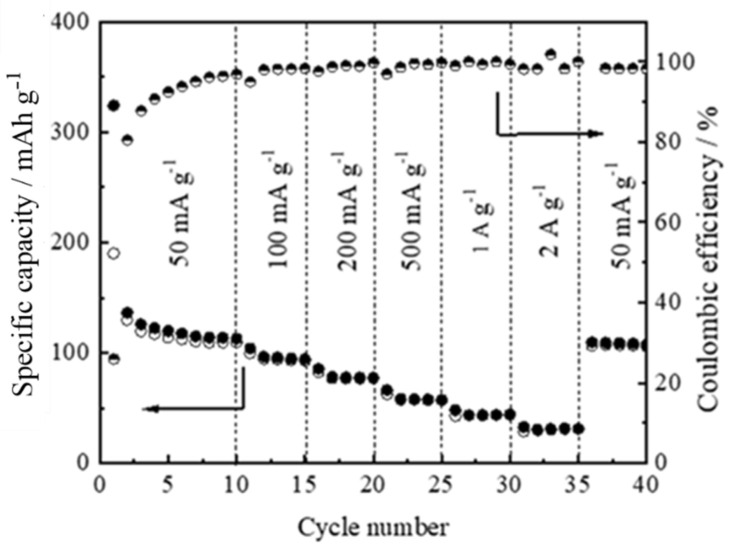
Rate performance of rGO.

To investigate the performance of Bi_2_O_3_@rGO composites and bare Bi_2_O_3_ particles for SIBs, the cycle performance and rate capability are evaluated. The Bi_2_O_3_@rGO exhibits a reversible discharge capacity of 160.9 mAh g^−1^ after 100 cycles (Figure 4c), which is higher than those of the Bi_2_O_3_ (<50 mAh g^−1^) and rGO electrode (Figure A5, ~100 mAh g^−1^). It has also been reported that the introduction of a carbon-based material can more effectively enhance the reversible capacity of the composite than bare Bi_2_O_3_ [16,31]. However, there are few discussions about the CE of Bi_2_O_3_-based materials. In this work, the initial CE of Bi_2_O_3_@rGO is 42.51%, due to the formation of SEI and electrolyte decomposition. The CE rapidly increases to 90.0% after 3 cycles and stabilizes at >95% after 6 cycles, whereas the CE of Bi_2_O_3_ is 89.2% even after 13 cycles. Figure 4d describes the rate capability of Bi_2_O_3_@rGO and Bi_2_O_3_. The composite electrode delivers specific capacities of 162, 135, 119, 105, 94 and 83 mAh g^−1^ at 0.05, 0.1, 0.2, 0.5, 1, and 2 A g^−1^, respectively. As seen, the capacity recovers to 141 mAh g^−1^ when the current density reduces to 0.05 A g^−1^. In contrast, the capacity of bare Bi_2_O_3_ quickly drops to about 10 mAh g^−1^ at 0.2 A g^−1^, due to the deteriorated electron transfer at high current densities and the unconstrained volume expansion of Bi_2_O_3_ without the presence of rGO. The capacities of pure rGO (Figure A6) are also much lower than those of the composite at all current densities. Furthermore, the composite shows good cycling stability at higher current densities, delivering stable capacities of 105 and 97.98 mAh g^−1^ at 0.2 and 1 A g^−1^, respectively (Figure 4e,f), completely outperforming the Bi_2_O_3_.

**Figure 5:**
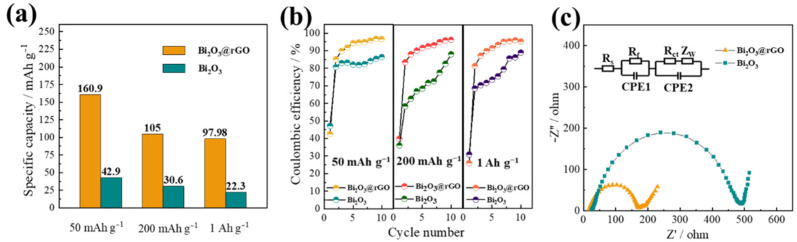
The comparison of (**a**) discharge capacities and (**b**) CEs of Bi_2_O_3_@rGO and Bi_2_O_3_. (**c**) Nyquist plots of Bi_2_O_3_@rGO and Bi_2_O_3_ after 100 cycles at 50 mA g^−1^.

Figure 5a compares the discharge capacities of Bi_2_O_3_@rGO and Bi_2_O_3_ electrodes after 100 cycles at different current densities. It is evident that the Bi_2_O_3_@rGO electrode exhibits much better cycle performance than the Bi_2_O_3_ electrode. Furthermore, the CEs in the first 10 cycles (Figure 5b) clearly shows that the CE of the Bi_2_O_3_@rGO electrode increases much more rapidly than that of the Bi_2_O_3_ at all tested current densities. Meanwhile, the CE also remains higher for the former than the latter and is responsible for the better cycling stability of the former. To further explicate the improved electrochemical performance of Bi_2_O_3_@rGO, the Nyquist plots of the two samples are presented in Figure 5c. The Nyquist plots of Bi_2_O_3_@rGO and Bi_2_O_3_ have similar shapes, consisting of a semicircle in the high-frequency region and a straight line in the low-frequency region. Obviously, Bi_2_O_3_@rGO composites have a smaller charge-transfer resistance (R_ct_) value than bare Bi_2_O_3_, confirming that the incorporation of Bi_2_O_3_ nanoparticles into rGO layers is beneficial to improve the rapid electron transfer during cycling.

**Figure 6:**
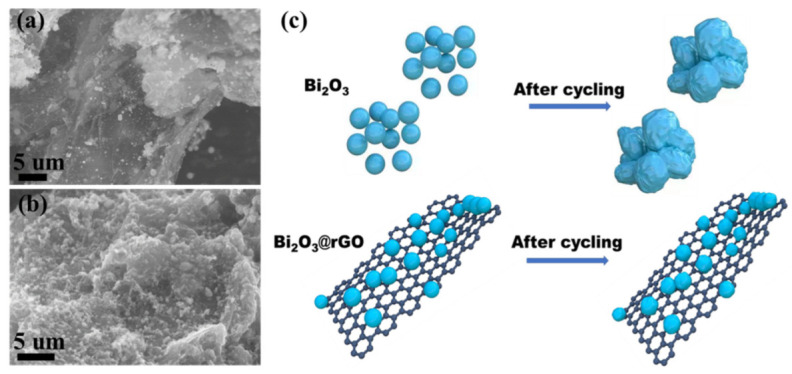
SEM images of Bi_2_O_3_@rGO (**a**) before cycling and (**b**) after cycling. (**c**) Schematic of the deformation of the two samples after cycling.

To further confirm the cycle stability of the composite, we have taken SEM images of Bi_2_O_3_@rGO before and after 100 cycles at 50 mA g^−1^ (Figure 6a,b). Figure 6b shows that Bi_2_O_3_ nanoparticles are still uniformly anchored on rGO layers with strong contact and kept clear shape. Schematic illustration of the deformation of Bi_2_O_3_ and Bi_2_O_3_@rGO after cycling is displayed in Figure 6c. In the absence of rGO layers, Bi_2_O_3_ particles deform after cycling, which could lead to unstable coulombic efficiency as well as inferior cycling stability. The presence of rGO layers can suppress volume expansion of Bi_2_O_3_ nanoparticles during cycling and reduce particle aggregation, contributing to better CE and cycle performance of Bi_2_O_3_@rGO.

## 4. Conclusions

In summary, a Bi_2_O_3_@rGO heterostructure was prepared and applied as a composite anode for SIBs. The anode exhibited a reversible capacity of 160.9 mAh g^−1^ after 100 cycles at 50 mA g^−1^ and its CE rapidly increased to >90% after 3 cycles. Both the cycle performance and CE are much enhanced compared with bare Bi_2_O_3_ particles used as an anode. The enhancement is attributed to rGO layers which act as a conductive template, shorten the diffusion length of Na-ion, and effectively suppress the agglomeration of Bi_2_O_3_ nanoparticles during the charge-discharge process. Our method demonstrates a synergy between the active material and the 2D conductive template and has the potential to be applied for a wide range of SIB electrode materials.

## Data Availability

The data presented in this study are contained within the article.
